# The Relationship Between Obesity, Overweight, and the Prevalence of Depression and Anxiety Among University Students: Evidence from a Nationally Representative Cross-Sectional Study in Greece

**DOI:** 10.3390/diseases14040136

**Published:** 2026-04-08

**Authors:** Olga Alexatou, Konstantinos Papadimitriou, Exakousti-Petroula Angelakou, Sousana K. Papadopoulou, Myrsini Pappa, Apostolia Ntovoli, Aspasia Serdari, Konstantina Apostolidou, Theophanis Vorvolakos, Constantinos Giaginis

**Affiliations:** 1 Department of Food Science and Nutrition, School of Environment, University of Aegean, 81400 Myrina, Lemnos, Greece; fnsd23003@fns.aegean.gr (O.A.); pangelakou@fns.aegean.gr (E.-P.A.); fnsm23028@fns.aegean.gr (M.P.); 2 Department of Nutritional Sciences and Dietetics, School of Health Sciences, International Hellenic University, 57400 Thessaloniki, Greece; kpapadimitriou@ihu.gr (K.P.); sousana@the.ihu.gr (S.K.P.); nantia@otenet.gr (K.A.); 3 Department of Medicine, School of Health Sciences, Democritus University of Thrace, 68100 Alexandroupolis, Greece; 4 Department of Physical Education and Sport Sciences, Frederick University, 3080 Limassol, Cyprus; antovoli@phed.auth.gr; 5 Department of Psychiatry, School of Medicine, Democritus University of Thrace, 68100 Alexandroupolis, Greece; aserntar@med.duth.gr (A.S.); tvorvola@med.duth.gr (T.V.)

**Keywords:** obesity, overweight, depression, anxiety, lifestyle factors, sociodemographic factors, mediterranean diet

## Abstract

Background and Objectives: Rates of obesity have been consistently increasing in recent years across all age groups, with a notable rise among young people. Obesity represents a persistent inflammatory condition and a key contributor to various chronic health problems, such as cardiovascular disorders, metabolic abnormalities, cancer, and psychological conditions. The move from high school to university is a transitional phase accompanied by specific pressures that can affect both body weight control and mental health in students. This cross-sectional investigation aimed to investigate potential associations between excess weight and the presence of depressive and anxiety symptoms in university populations. Methods: This cross-sectional analysis included 5298 students enrolled at universities across ten geographic areas of Greece. Participants filled out questionnaires concerning demographic information and lifestyle behaviors. Levels of depression and anxiety were measured using the Beck Depression Inventory (BDI-II) and the short form of the State Anxiety Inventory (STAI-6), respectively. Measurements of height and body weight were obtained to compute Body Mass Index (BMI). Results: The presence of overweight or obesity among students was significantly and independently related to female sex, urban residence, living independently, tobacco use, and lower academic performance (*p* = 0.0103, *p* = 0.0102, *p* = 0.0203, *p* = 0.0075, and *p* = 0.0168, respectively). Individuals reporting insufficient physical activity had 85% higher odds of being overweight or obese (*p* = 0.0068). Similarly, participants experiencing depressive or anxious symptomatology had more than double odds of excess body weight compared with those without such symptoms (*p* = 0.0015 and *p* = 0.0012, respectively). Furthermore, poor Mediterranean diet adherence was linked to more than a twofold increase in the odds of overweight or obesity (*p* = 0.0005). Conclusions: These findings offer considerable evidence that symptoms of depression and anxiety may serve as significant contributors to the development of overweight and obesity among university students. Additional longitudinal studies are strongly encouraged to substantiate these observations.

## 1. Introduction

Overweight and obesity involve excessive adipose tissue accumulation, with obesity recognized as a chronic inflammatory disorder that can significantly impair health and well-being [1,2]. They increase the risk of type 2 diabetes [3], cardiovascular diseases [4], various cancers [5], reduced bone strength [6], reproductive dysfunction [7], inflammatory bowel diseases [8,9], and lower quality of life, including poor sleep [10] and reduced mobility [11]. They are commonly assessed using body mass index (BMI), calculated from weight and height, often complemented by waist circumference to evaluate abdominal fat distribution [12]. Although BMI thresholds for children and adolescents vary by age [13] and sex [14], adult data highlight the growing global burden of obesity. Globally, overweight and obesity have increased sharply since 1990, affecting over 2 billion adults in 2021 and projected to surpass 3.8 billion by 2050 [15]. The rise is especially pronounced in Oceania, North Africa, and the Middle East, while sub-Saharan Africa may see a 254.8% increase by 2050. Socioeconomic inequalities further influence these trends [15]. Socioeconomic strain is strongly associated with obesity prevalence, though effects vary across countries and genders [16]. Additionally, urban living, tobacco use, low physical activity, stress, and poor sleep are linked to higher overweight and obesity rates among young adults [17]. Overall, these findings highlight the need for targeted, multisectoral strategies to address the growing global impact of obesity.

Obesity, beyond its clearly established metabolic and physical health outcomes, has increasingly been recognized as a significant contributor to a wide array of psychiatric and psychological disorders [18]. A considerable volume of evidence points to a strong, reciprocal relationship between obesity and depression. Prospective studies have documented odds ratios (ORs) ranging from 1.21 to 5.8 for developing depression among individuals with obesity, and from 1.18 to 3.76 for the opposite direction of association, with this effect being especially pronounced in women [19]. Furthermore, obesity has been linked to greater vulnerability to anxiety, stress-related disorders, and eating disorders, although these associations are typically described as modest to moderate in strength [19,20]. Leutner et al. further highlight that obesity commonly precedes the onset of several psychiatric conditions, including psychosis-spectrum disorders, personality disorders, and anxiety disorders across all age groups, with particularly elevated susceptibility among younger females [20]. A number of region-specific investigations reinforce these conclusions. For example, AlQahtani et al. reported meaningful correlations between obesity and increased levels of depression, anxiety, and stress among male university students in Saudi Arabia [21]. Similarly, Hossain et al. observed a strong positive association between obesity and the severity of depressive, anxiety-related, and stress symptoms in Bangladeshi university students, emphasizing the necessity for more detailed clinical evaluation [22]. In addition, Emon et al. examined the psychosocial impacts of obesity and found a strong negative relationship between BMI and both self-esteem and academic performance; students classified as overweight or obese were more than seven times more likely to report low self-esteem compared with their normal-weight peers [23].

Students in higher education are showing a growing exposure to mental health problems, notably depressive symptoms, anxiety, and stress. In a recent study, increased mean values were recorded for depression, anxiety, and stress with reported prevalence levels of 75%, 88.4%, and 84.4% [24]. These outcomes imply that manifestations of anxiety and stress—particularly within the moderate to extremely severe categories—were more prevalent than those of depression. This pattern is in line with previous research demonstrating that emotional and psychological burden is widespread across university settings. For instance, Bayram and Bilgel identified moderate or higher degrees of depression, anxiety, and stress in 27.1%, 47.1%, and 27% of their participants, respectively, with elevated levels noted among women and students in the early stages of their academic programs [25]. Liu et al. underscored the inverse connection between psychological well-being and affective disorders, indicating that abilities such as emotional regulation and self-acceptance may serve as protective buffers against depression and anxiety [26].

Mofatteh et al. delineated six principal categories of risk factors—psychological, academic, biological, lifestyle, social, and financial—that exert a substantial impact on students’ mental well-being [27]. Excessive smartphone use [28], insufficient sleep [29], academic stressors [30], and limited emotional coping capacities have been consistently associated with heightened levels of anxiety and depression [27]. Moreover, Saeed et al. reported that demographic and structural factors, including study system, gender, age, and living arrangements, significantly influence mental health outcomes [31]. The above findings underscore the critical need for early preventive interventions, targeted mental health support, and policy initiatives aimed at mitigating the psychosocial challenges faced by university students and promoting resilience during this formative developmental period.

Nonetheless, empirical research investigating the relationship between overweight and obesity and the prevalence of depression and anxiety in university populations remains limited, whereas no data are currently available for Greek university students. Moreover, the prevalence of obesity and overweight in Greek young people has been considered among the highest in the European countries as well as worldwide [32,33]. Moreover, several studies have shown that the incidence of obesity and overweight in university students is alarmingly high (approximately close to 30%) compared to other population categories [34,35]. In this aspect, the present cross-sectional study aims to evaluate for the first time the potential relationship between overweight/obesity and depression and anxiety among university students in Greece. Additionally, the present study aims to assess potential links between overweight and obesity and various socio-demographic (e.g., age, nationality, gender, financial status, etc.) and lifestyle factors (e.g., living status, smoking status, employment status, physical activity, etc.). Another purpose of the current study is to investigate whether a potential relationship between overweight/obesity and depression and anxiety could be independent of relevant confounding factors which could affect the above-mentioned relationship.

## 2. Methods

### 2.1. Study Population

**Figure 1:**
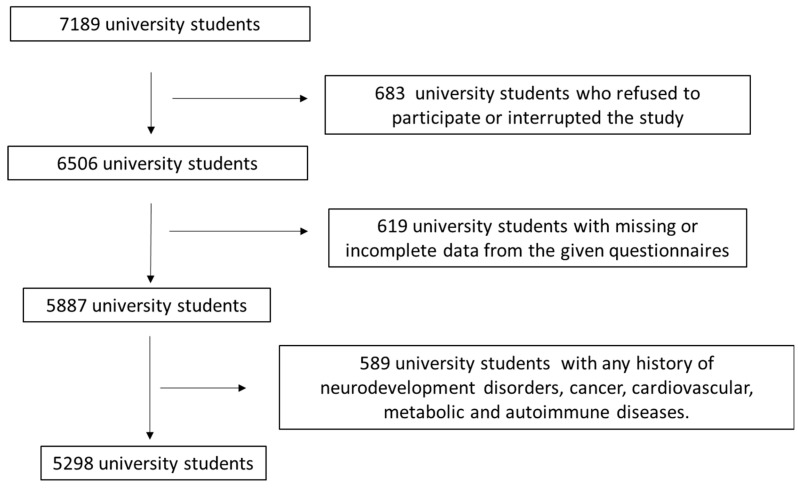
Flow chart diagram for study population enrollment.

This research initially enrolled 7189 university students from 10 distinct Greek regions, including both rural and urban areas (Athens, Thessaloniki, Larissa, Kalamata, Kavala, Korinthos, Alexandroupolis, Patra, South and North Aegean). Only students who were actively registered at Greek universities were eligible for participation. Recruitment for the survey occurred between March 2021 and October 2024. Figure 1 presents the flowchart illustrating participants’ enrollment. Relevant inclusion and exclusion criteria were applied, as described in Figure 1. Ultimately, 5298 active university students were included, resulting in a final response rate of 73.7%.

The study protocol received approval from the Ethics Committee of the University of the Aegean (ethics approval code: no 21/11.10.2017, approval date: 11 October 2017), in line with World Health Organization standards (52nd WMA General Assembly, Edinburgh, Scotland, 2000). All participants’ information was maintained confidentially. None of the participants reported a history of chronic conditions, including cardiometabolic diseases, mental disorders, or malignant tumors. Additionally, all enrolled students were informed about the objectives of the survey and provided written consent. Sample size calculations were performed using PS: Power and Sample Size estimator software.

### 2.2. Assessment of Sociodemographic and Anthropometric Factors

Appropriate questionnaires were utilized to gather data on the sociodemographic and lifestyle attributes of the participating university students (e.g., age, gender, nationality, residence, family financial situation, living conditions, parents’ marital status, smoking patterns, and employment status). Furthermore, all participants were actively enrolled in Greek universities, and information regarding their academic achievements and field of study was collected. The sociodemographic and lifestyle data were obtained through individual interviews conducted between the students and professional nutritionists and dietitians to reduce recall bias.

Family financial situation was categorized according to annual household income: low for yearly income ≤ 10,000€, moderate for income > 10,000€ and ≤20,000€, and high for income > 20,000€. Academic performance was classified based on the average course grades at the time of the survey: low for grades ranging from 5.0 to 6.49, moderate for grades between 6.5 and 8.49, and high for grades between 8.5 and 10.0, following the official criteria of the Minister of Education of Greece.

Participants’ body weight and height were measured during the survey to determine Body Mass Index (BMI). Weight was assessed using a Seca scale (Seca, Hanover, MD, USA) without shoes and recorded to the nearest 100 g, while height was measured with a portable stadiometer (GIMA Stadiometer 27335) without shoes and recorded to the nearest 0.1 cm [36,37]. The WHO guidelines were used to categorize the students as normal weight, overweight, or obese [36,37]. All continuous data were transformed into categorical data mainly to simplify interpretation, support decision-making, enable certain statistical analyses and highlight meaningful thresholds. Moreover, from a clinical point of view, transforming continuous data into categorical variables is often justified for practical, diagnostic, and decision-making purpose.

### 2.3. Assessment of Physical Activity, Depression, Anxiety and Nutritional Habits

The level of physical activity among university students was evaluated using the International Physical Activity Questionnaire (IPAQ). Students indicated the amount of exercise they usually performed during a typical week. This questionnaire is extensively utilized globally and classifies physical activity into low, moderate, or high categories [38]. The IPAQ has been tested in both high-income and middle-income countries, showing strong reliability and acceptable validity. In summary, the IPAQ aims to combine vigorous, moderate, and walking activities over the previous seven days to calculate an overall physical activity score (PA score), expressed in MET-minutes per week (MET·min·wk^−1^) [38].

The Beck Depression Inventory (BDI-II) was employed to evaluate depression in university students. The tool contains 21 groups of statements and is recognized as one of the most widely applied psychometric instruments for measuring the severity of depressive symptoms [39]. The BDI-II addresses items related to depression, including emotional experiences such as hopelessness and irritability, cognitive patterns like self-blame or feelings of punishment, as well as physical manifestations, including fatigue, weight loss, and reduced sexual interest [39]. The BDI-II is regarded as a reliable psychometric assessment, demonstrating high consistency in differentiating depressed from non-depressed individuals, along with strong content and construct validity. According to available psychometric data, the BDI-II represents a cost-effective questionnaire for evaluating depressive symptom severity and exhibits broad applicability [39]. Participants rate the severity of their symptoms using a four-point ordinal response scale (0–3), yielding aggregate scores ranging from 0 to 63, with higher scores indicating more pronounced depressive symptomatology. The Greek version of the BDI-II demonstrates robust psychometric properties, including high internal reliability (Cronbach’s α = 0.93) and adequate validity in both clinical and non-clinical samples [40]. In the current study sample, the BDI-II showed acceptable internal reliability (Cronbach’s α = 0.847), further supporting its appropriateness for assessing depressive symptoms in a university student population.

The six-item version of the State subscale of the anxiety inventory (STAI-6) questionnaire was employed to measure anxiety among the enrolled students. STAI-6 is an abbreviated form of the state scale that includes six questions and shows strong internal consistency and validity [41]. It was created for use in contexts where administration of the complete instrument is not feasible and generates scores that are comparable to those obtained from the full version [41]. The instrument assesses key affective components related to anxiety, such as sensations of calmness, relaxation, tension, distress, worry, and positive mood. Participants evaluate each item based on their immediate emotional state. To maintain consistency with the full STAI, raw scores were transformed into the standard STAI scale (range: 20–80), enabling interpretation according to established clinical benchmarks. According to previously validated guidelines, scores between 34 and 37 reflect typical anxiety levels, whereas scores of 38 or higher indicate increased anxiety symptomatology [42,43]. In the present sample, the scale exhibited acceptable internal reliability (Cronbach’s α = 0.805), supporting its suitability for measuring maternal anxiety within this group.

Mediterranean Diet (MD) adherence in the participating university students was evaluated with the KIDMED questionnaire [44]. This tool is among the most commonly used instruments for estimating MD adherence in children, adolescents, and young adults. It demonstrates high reliability and validity and consists of 16 items designed to assess eating behaviors. Each item is answered with “yes” or “no” and scored from −1 to +1. Twelve items receive a positive value, whereas four items receive a negative value. The total KIDMED score ranges from 0 to 12 and is categorized as follows: ≥8 points, high adherence; 4–7 points, moderate adherence; and ≤3 points, low adherence to the MD [44].

All previously mentioned lifestyle parameters—such as physical activity, depressive symptoms, anxiety, and dietary patterns—were obtained through in-person interviews between university students and trained staff in order to reduce recall bias.

### 2.4. Statistical Analysis

Student’s *t*-test was applied to examine continuous variables that followed a normal distribution. The Kolmogorov–Smirnov test was performed to verify the normality of these continuous data. The chi-square test was utilized for the analysis of categorical variables. Normally distributed continuous variables were expressed as mean ± standard deviation (SD). Categorical data were described as absolute counts or percentage frequencies. Multivariable binary logistic regression analysis was carried out to assess whether overweight/obesity in university students was independently associated with sociodemographic and lifestyle characteristics, including depression and anxiety, with adjustment for potential confounding factors. Odds ratios were used as an effect size for categorical data, which quantifies the magnitude of a relationship or difference, independent of sample size. All statistical procedures were performed using Statistica 10.0 software, Europe (Informer Technologies, Inc., Hamburg, Germany).

## 3. Results

### 3.1. Descriptive Statistics of the Study Population

The summary statistics for the included university students are displayed in Table S1 (Supplementary Materials). Altogether, 5298 students were included in the current research, presenting a mean age of 21.4 ± 2.5 years. Of the total sample, 52.0% were female and 82.0% held Greek citizenship. Moreover, 58.6% of the students were living in urban areas across Greece, while 41.4% were residing in rural locations. Regarding economic status, 43.1% of participants were classified as having a low financial standing, 37.4% were categorized as having a moderate financial standing, and 19.5% were characterized by a high financial standing. Concerning housing status, 53.9% of the students were living with their families, whereas 46.1% were living on their own. Lastly, 32.5% of the surveyed students reported having divorced parents, and 39.2% stated that they were regular smokers.

Furthermore, 56.4% of the registered university students pursued biomedical fields. Additionally, 41.1% of the surveyed university students achieved good academic results, 37.3% attained very good results, and only 21.6% reached excellent results. Moreover, 30.7% of the surveyed university students were employed. In conclusion, 14.4% of the students were identified as overweight and 8.9% as obese.

Concerning lifestyle characteristics, 38.3% of the surveyed university students demonstrated low physical activity levels, 35.2% indicated moderate activity, and merely 26.5% demonstrated high activity levels. Furthermore, 32.5% of the surveyed university students experienced depressive symptoms, and 34.5% reported anxiety-related manifestations. Lastly, 47.4% of the surveyed university students were determined to have low adherence to the MD, 34.4% exhibited moderate adherence to the MD, and only 18.2% demonstrated high adherence to the MD.

### 3.2. Relationships Between BMI and Sociodemographic and Lifestyle Factors

Within the cross-tabulation procedure, links between university students’ BMI and the collected variables were assessed (Table 1). Excess body weight and obesity occurred significantly more frequently in female students than in male students (*p* ˂ 0.0001). Students living in metropolitan settings had a markedly greater prevalence of overweight or obesity than those residing in rural settings (*p* ˂ 0.0001). Normal weight participants indicating a higher household economic status compared with overweight participants; however, this trend was not observed when comparing normal weight and overweight participants with obese participants (*p* = 0.0012). Those living on their own showed a greater likelihood of overweight or obesity than peers living with family members (*p* = 0.0023).

Students with divorced parents demonstrated increased rates of overweight or obesity compared with students whose parents were married (*p* ˂ 0.0001). University students who smoked had a higher probability of being overweight or obese than non-smoking students (*p* ˂ 0.0001). Academic outcomes were significantly poorer among overweight or obese students (*p* = 0.0027). Students reporting low physical activity were more commonly overweight or obese than those reporting moderate or high activity (*p* ˂ 0.0001). Greater proportions of overweight and obesity were also observed among students with perceived stress symptoms (*p* ˂ 0.0001). Likewise, overweight and obesity appeared significantly more frequently in students presenting anxiety symptoms (*p* ˂ 0.0001). Lastly, students with lower adherence to the Mediterranean diet (MD) were more frequently overweight or obese than those with higher adherence levels (*p* ˂ 0.0001).

### 3.3. Multivariate Binary Logistic Regression Analysis for BMI

In multivariate binary logistic regression analysis, students’ gender, type of residence, living situation, smoking status, academic performance, physical activity, depression, anxiety, and adherence to the MD were all significantly and independently correlated with BMI (Table 2, *p* ˂ 0.05). Notably, female students had a 25% higher prevalence of overweight or obesity compared with male students (*p* = 0.0103). Students living in urban areas exhibited 45% greater odds of being overweight or obese compared with those in non-urban areas (*p* = 0.0102). Those residing alone had a 38% higher likelihood of being overweight or obese than peers living with their family (*p* = 0.0203).

Smokers showed a 71% higher incidence of overweight or obesity relative to non-smokers (*p* = 0.0075). Students with lower academic achievement had 22% greater odds of being overweight or obese (*p* = 0.0168). Reduced physical activity levels were linked to an 85% increased risk of overweight or obesity (*p* = 0.0068). Students exhibiting symptoms of depression or anxiety were more than twice as likely to be overweight or obese (*p* = 0.0015 and *p* = 0.0012, respectively). Furthermore, students with poor adherence to the MD had over double the likelihood of being affected by overweight or obesity (*p* = 0.0005).

## 4. Discussion

The present survey found that 14.4% of university students were overweight and 8.9% obese. Depressive symptoms were reported by 32.5% and anxiety by 34.5%. Overweight and obesity were more common among female students, who more often reported lower economic status (43.1%). However, students from higher-income families were also more likely to be overweight or obese. Increased prevalence was observed among those living alone (46.1%), with divorced parents (32.5%), and regular smokers (39.2%), as well as among urban residents. Low physical activity (38.3%) was strongly associated with excess weight, increasing the likelihood by 85%. Poor adherence to the Mediterranean diet more than doubled the odds, while depressive and anxiety symptoms were linked to a two-fold or greater risk. Overall, overweight and obesity were influenced by gender, socioeconomic and family factors, mental health, lifestyle behaviors, and diet.

Depression and anxiety cases have increased markedly, especially after COVID-19 [45]. In our study, prevalence rates were lower (32.5% for depression; 34.5% for anxiety) compared with findings by Amaro et al. (61.2% and 75%) and Lun et al. (68.5% and 54.4%) [46,47]. These differences may reflect regional, methodological, or temporal factors [48]. However, our results align with Li et al.’s meta-analysis reporting global rates of 33.6% for depression and 39.0% for anxiety [49]. This suggests our sample reflects global averages rather than extreme local estimates, underscoring the influence of contextual factors such as demographics, socioeconomic status, academic pressure, and healthcare access on mental health outcomes [50].

While our study assessed the prevalence of depression and anxiety, Liu and Wang and other researchers examined causal links with university life satisfaction, finding that depression—but not anxiety—predicts later dissatisfaction [47,51], and that greater satisfaction reduces both symptoms over time [52]. Our cross-sectional design cannot determine temporal effects, though reported rates may reflect underlying stressors. Other studies identify psychosocial determinants such as socioeconomic status [53], physical activity [54], and academic achievement [55] as risk or protective factors [46,47]. These findings emphasize the need for multifaceted assessments and support future research on targeted mental health interventions in universities.

A meta-analysis of 32 cross-sectional studies reported depression prevalence ranging from 12.1% to 77.1%, with a pooled rate of 34.70%, consistent with our findings [56]. Another systematic review and meta-analysis across 20 low- and middle-income countries found an overall incidence of 24.4%, with no significant differences by study design or other methodological factors [57]. Additionally, a Nigerian study linked obesity to higher depression in male students [58], while research among female undergraduates showed greater depression among overweight and obese students compared to those of normal weight [59].

A cross-sectional study of 147 undergraduates found higher anxiety associated with overweight/obesity and elevated body fat [60]. Similarly, research among 610 medical students showed that overweight/obese individuals were more prone to burnout and internet addiction, linked to greater depression, anxiety, and stress [61]. Among 1381 college students, anxiety prevalence was 30.1%, with a significant association between obesity and anxiety, particularly in males [62]. A meta-analysis of 25 studies likewise reported higher anxiety rates in overweight and obese individuals compared with those of normal weight [63].

The advantages of our study compared with prior research include a large participant cohort and a thorough, multifaceted evaluation of possible factors contributing to depression and anxiety. Socioeconomic status, levels of physical activity, nutritional patterns, and experiences of abuse may influence depressive and anxiety symptoms and merit further investigation in future studies. Moreover, we carried out in-person interviews between participants and trained personnel, which helps mitigate recall bias typically associated with self-reported data. In addition, we utilized standardized, validated questionnaires to enhance both reliability and accuracy of the collected information.

Nonetheless, several limitations of the study should be recognized. First, the research was conducted solely among university students attending Greek universities, restricting the generalizability of the findings to students in other countries. Second, although face-to-face interviews were used to gather data, the information remains self-reported, so recall bias cannot be fully excluded. Third, despite implementing multivariate analyses that accounted for multiple potential confounding variables, unmeasured confounders may still have influenced the results. Fourth, even if the classification of academic performance based on Greek grading system could be a strength of the present study, it may not be generalizable concerning the university students of other countries. Fifth, although the odds ratios and confidence intervals in logistic regression analysis seems impressive, the wide confidence intervals for some variables (e.g., age, nationality) may suggest instability. Finally, the cross-sectional nature of the study prevents drawing conclusions about causal relationships between overweight/obesity and depression or anxiety among university students.

## 5. Conclusions

This is among the first cross-sectional studies examining the relationship between overweight and obesity and the occurrence of depression and anxiety among university students in Greece, while accounting for a wide range of potential confounding factors. The findings revealed significant associations between overweight and obesity and increased levels of depression and anxiety in university students, independent of multiple sociodemographic and lifestyle variables. From a clinical point of view, the high prevalence of obesity/overweight and its relationship with depression/anxiety may increase the risk for the development of various comorbidities (e.g., cardiovascular diseases, metabolic disorders, anorexia nervosa and bulimia, etc.) in the next stages of the university students’ life. Nevertheless, longitudinal research is strongly encouraged to confirm these results. Public health policies and nutrition education initiatives should be implemented to address the high rates of overweight and obesity, while concurrently aiming to reduce the prevalence of depression and anxiety among university students. Additionally, promoting healthy dietary patterns and providing nutritious food options in university dining facilities in Greece is recommended.

## Figures and Tables

**Table 1:** Comparison of the distribution of covariates (e.g., sociodemographic and lifestyle factors) within the categories of the variable of interest (e.g., BMI categories; normal weight, overweight and obesity).

Characteristics (n = 5298)	BMI			
	Normal Weight 4064 (76.7%)	Overweight 760 (14.4%)	Obesity 474 (8.9%)	*p*-Value
Age (mean ± SD; years)	21.4 ± 2.4	21.5 ± 5	21.3 ± 2.5	*p* = 0.2491
Gender (n, %)				*p* ˂ 0.0001
Male	2199 (54.1%)	371 (48.8%)	187 (39.4%)	
Female	1865 (45.9%)	389 (51.2%)	287 (60.6%)	
Nationality (n, %)				*p* = 0.0854
Greek	3324 (81.8%)	661 (87.0%)	358 (75.5%)	
Other	740 (18.2%)	99 (13.0%)	116 (24.5%)	
Type of residence (n, %)				*p* ˂ 0.0001
Urban	2516 (61.9%)	436 (57.4%)	108 (22.7%)	
Rural	1548 (38.1%)	324 (42.6%)	319 (67.3%)	
Family financial level				*p* = 0.0012
Low	1813 (44.6%)	325 (42.8%)	145 (30.6%)	
Moderate	1448 (35.6%)	320 (42.1%)	213 (44.9%)	
High	803 (19.8%)	115 (15.1%)	116 (24.5%)	
Living status (n, %)				*p* = 0.0023
Living with family	2280 (56.1%)	421 (55.4%)	154 (32.5%)	
Living alone	1784 (43.9%)	339 (44.6%)	320 (67.5%)	
Parents marital status				*p* ˂ 0.0001
Married	2835 (69.8%)	510 (67.1%)	232 (48.9%)	
Divorced	1229 (30.2%)	250 (32.9%)	242 (51.1%)	
Smoking status				*p* ˂ 0.0001
No smokers	2571 (63.3%)	417 (54.9%)	233 (49.2%)	
Regular smokers	1493 (36.7%)	343 (45.1%)	241 (50.8%)	
Type of Studies				*p* = 0.6981
Biomedical studies	2304 (56.7%)	424 (55.8%)	260 (54.9%)	
Other studies	1760 (43.3%)	336 (44.2%)	214 (45.1%)	
Academic performance				*p* = 0.0027
Good	1585 (39.0%)	298 (39.2%)	293 (61.8%)	
Very good	1548 (38.1%)	339 (44.6%)	90 (19.0%)	
Excellent	931 (22.9%)	123 (16.2%)	91 (19.2%)	
Employment status				*p* = 0.0872
No employee	2831 (69.7%)	539 (70.9%)	302 (63.7%)	
Employee	1233 (30.3%)	221 (29.1%)	172 (36.3%)	
Physical activity (n, %)				*p* ˂ 0.0001
Low	1416 (38.8%)	376 (49.5%)	235 (49.6%)	
Moderate	1524 (37.5%)	206 (27.1%)	134 (28.3%)	
High	1124 (27.7%)	178 (23.4%)	105 (22.1%)	
Depression (n, %)				*p* ˂ 0.0001
No	3117 (76.7%)	265 (34.9%)	197 (41.6%)	
Yes	947 (23.3%)	495 (65.1%)	277 (58.4%)	
Anxiety (n, %)				*p* ˂ 0.0001
No	2911 (71.6%)	357 (47.0%)	205 (43.2%)	
Yes	1153 (28.4%)	403 (53.0%)	269 (56.8%)	
KIDMED (n, %)				*p* ˂ 0.0001
Low	1562 (38.4%)	471 (62.0%)	317 (66.9%)	
Moderate	1639 (40.3%)	187 (24.6%)	83 (17.5%)	
High	863 (21.2%)	102 (13.4%)	74 (15.6%)	

**Table 2:** Multivariate analysis for BMI of the study population.

Characteristics	BMI (Obesity & Overweight vs. Normal Weight)	
	OR * (95% CI **)	*p*-Value
Age (Over/Below mean value)	1.12 (0.20–2.15)	*p* = 0.3682
Gender (Male/Female)	0.75 (0.60–0.94)	*p* = 0.0103
Nationality (Greek/Other)	0.95 (0.16–1.87)	*p* = 0.4881
Type of residence (Urban/Rural)	1.45 (1.14–1.78)	*p* = 0.0102
Family financial status (Low/Moderate & High)	0.89 (0.23–1.52)	*p* = 0.1954
Living status (Living alone/Living with family)	1.38 (0.89–1.85)	*p* = 0.0203
Parents marital status (Divorced/Married)	1.22 (0.46–1.99)	*p* = 0.2542
Smoking status (Regular smokers/No smokers)	1.71 (1.52–1.98)	*p* = 0.0075
Type of Studies (Biomedical studies/Other studies)	0.98 (0.25–1.82)	*p* = 0.7463
Academic performance (Good/Very good & Excellent)	1.22 (0.84–1.67)	*p* = 0.0168
Employment status (No employee/Employee)	0.95 (0.23–1.82)	*p* = 0.3821
Physical activity (Low/Moderate & High)	1.85 (1.64–2.07)	*p* = 0.0068
Depression (Yes/No)	2.31 (2.15–2.48)	*p* = 0.0015
Anxiety (Yes/No)	2.46 (2.31–2.65)	*p* = 0.0012
KIDMED (Low/Moderate & High)	2.55 (2.42–2.69)	*p* = 0.0005

* Odds Ratio: OR. ** CI: Confidence Interval.

## Data Availability

Data is available upon request to the corresponding author.

## Supplementary Materials

The following supporting information can be downloaded at: https://www.mdpi.com/article/10.3390/diseases14040136/s1, Table S1: Descriptive statistics of the enrolled university students.
