# Supplementary material for: The Relationship Between Obesity, Overweight, and the Prevalence of Depression and Anxiety Among University Students: Evidence from a Nationally Representative Cross-Sectional Study in Greece
Source: Diseases. 2026 Apr 8;14(4):136. doi: 10.3390/diseases14040136 (PMC13114611; doi:10.3390/diseases14040136)
Supplement: Supplementary file 1 [file diseases-14-00136-s001.zip › diseases-4188604-supplementary.pdf]

Table S1. Descriptive statistics of the enrolled university students.

Characteristics (n=5298)	Descriptive statistics
**Age (mean±SD; years)**	21.4±2.5
**Gender (n, %)**	
Male	2541 (48.0%)
Female	2757 (52.0%)
**Nationality (n, %)**	
Greek	4343 (82.0%)
Other	955 (18.0%)
**Type of residence (n, %)**	
Urban	3107 (58.6%)
Rural	2191 (41.4%)
**Family financial level**	
Low	2283 (43.1%)
Moderate	1981 (37.4%)
High	1034 (19.5%)
**Living status (n, %)**	
Living with family	2855 (53.9%)
Living alone	2443 (46.1%)
**Parents marital status**	
Married	3577 (67.5%)
Divorced	1721 (32.5%)
**Smoking status**	
No smokers	3221 (60.8%)
Smokers	2077 (39.2%)
**Type of Studies**	
Biomedical studies	2988 (56.4%)
Other studies	2310 (43.6%)
**Academic performance**	
Good	2176 (41.1%)
Very good	1977 (37.3%)
Excellent	1145 (21.6%)
**Employment status**	

Employee	1626 (30.7%)
No employee	3672 (69.3%)
**Physical activity (n, %)**	
Low	2027 (38.3%)
Moderate	1864 (35.2%)
High	1405 (26.5%)
**BMI (n, %)**	
Normal weight	4064 (76.7%)
Overweight	760 (14.4%)
Obese	474 (8.9%)
**Depression (n, %)**	
No	3579 (67.5%)
Yes	1719 (32.5%)
**Anxiety (n, %)**	
No	3473 (65.5%)
Yes	1825 (34.5%)
**KIDMED (n, %)**	
Low	2511 (47.4%)
Moderate	1824 (34.4%)
High	963 (18.2%)
